# Transcriptional Responses Associated with Virulence and Defence in the Interaction between *Heterobasidion annosum s*.*s*. and Norway Spruce

**DOI:** 10.1371/journal.pone.0131182

**Published:** 2015-07-07

**Authors:** Karl Lundén, Marie Danielsson, Mikael Brandström Durling, Katarina Ihrmark, Miguel Nemesio Gorriz, Jan Stenlid, Frederick O. Asiegbu, Malin Elfstrand

**Affiliations:** 1 Department of Forest Mycology and Plant Pathology, Uppsala Biocenter, Swedish University of Agricultural Sciences, Uppsala, Sweden; 2 Chemistry, School of Chemical Science and Engineering, KTH Royal Institute of Technology, Stockholm, Sweden; 3 Department of Forest Sciences, University of Helsinki, Helsinki, Finland; Università Politecnica delle Marche, ITALY

## Abstract

*Heterobasidion annosum sensu lato* is a serious pathogen causing root and stem rot to conifers in the northern hemisphere and rendering the timber defective for sawing and pulping. In this study we applied next-generation sequencing to i) identify transcriptional responses unique to *Heterobasidion*-inoculated Norway spruce and ii) investigate the *H*. *annosum* transcripts to identify putative virulence factors. To address these objectives we wounded or inoculated 30-year-old Norway spruce clones with *H*. *annosum *and 454-sequenced the transcriptome of the interaction at 0, 5 and 15 days post inoculation. The 491860 high-quality reads were *de novo* assembled and the relative expression was analysed. Overall, very few *H*. *annosum* transcripts were represented in our dataset. Three *delta-12 fatty acid desaturase* transcripts and one *Clavaminate synthase-like* transcript, both associated with virulence in other pathosystems, were found among the significantly induced transcripts. The analysis of the Norway spruce transcriptional responses produced a handful of differentially expressed transcripts. Most of these transcripts originated from genes known to respond to *H*. *annosum*. However, three genes that had not previously been reported to respond to *H*. *annosum* showed specific induction to inoculation: an *oxophytodienoic acid–reductase (OPR)*, a *beta–glucosidase *and a *germin-like protein* (*GLP2*) gene. Even in a small data set like ours, five novel highly expressed Norway spruce transcripts without significant alignment to any previously annotated protein in Genbank but present in the *P*. *abies* (v1.0) gene catalogue were identified. Their expression pattern suggests a role in defence. Therefore a more complete survey of the transcriptional responses in the interactions between Norway spruce and its major pathogen *H*. *annosum *would probably provide a better understanding of gymnosperm defence than accumulated until now.

## Introduction

Norway spruce [*Picea abies* (L.) Karst.] is a dominating tree species in much of northern Eurasia. It relies on both induced and constitutive defences to restrict the spread of invading fungi and insects. The bark is the first line of defence that provide an efficient defensive barrier [1]. When this barrier-defence is breached, inducible defences remain essentially the only protection against invading pathogens [2]. It has been indicated that the defence responses in conifers lack specificity in their responses to pathogens [3–5]. The induced transcriptional responses to pathogenic fungi are similar to those induced by wounding in Norway spruce [1, 4, 6–8]. Both responses involve expression of genes encoding pathogenesis related- (PR) proteins and components of well-described defence signal transduction pathways but the expression levels are generally significantly higher after pathogen infection.

The single most important biotic stress factor to Norway spruce is the root rot fungus *Heterobasidion annosum sensu lato* (*s*.*l*.) [9]. *H*. *annosum s*.*l*. is a complex of five closely related species [10–12] that have different yet sometimes overlapping host ranges. In Scandinavia two species are present: *H*. *annosum sensu stricto* (*s*.*s*.) and *H*. *parviporum*, both pathogenic on Norway spruce [13]. Recently, the first genome of *H*. *annosum s*.*l*. was published, of the North American *H*. *irregulare* [14]. Transcriptional analyses of *H*. *irregulare* under different conditions suggest that living host tissues triggers an expanded metabolic repertoire involving genes associated with, for example, toxin production, pectin degradation, protection against plant defences, handling of low oxygen pressure and other abiotic stresses [14].

*H*. *annosum s*.*l*. causes root and stem rot and renders the timber defective for sawing and pulping. *H*. *annosum s*.*l*. infection also reduces the tree’s growth and increases the risk of wind-throw. Economic losses to the Scandinavian forest industry attributed to *H*. *annosum s*.*l*. infections exceed € 90 million annually [9]. Identification of resistant host genotypes and understanding the mechanisms behind such resistance would enable selection of more resistant host material for replanting, which could reduce the economic losses. In an earlier study of the transcriptional responses of genes in the phenylpropanoid biosynthetic pathway in the Norway spruce genotypes we showed that those genes were highly induced in response to wounding and infection by *H*. *annosum s*.*s*. [1]. In the current study, we expanded the analysis of the responses in these Norway spruce genotypes beyond genes in the phenylpropanoid pathway. Our objectives were to i) identify transcriptional responses unique to *Heterobasidion*-inoculated Norway spruce and ii) investigate the *H*. *annosum s*.*s*. transcripts and compare them to the expression patterns predicted from *H*. *irregulare* in Pine bark [14] to identify common virulence factors in *H*. *annosum s*.*l*.

## Material and Methods

### Plant material and sampling

In this study, we used 30-year-old trees of eight Norway spruce genotypes: S21K7822405, S21K7825237, S21K7827398, S21K7828590 S21K7823178, S21K7823340, S21K7825278 and S21K7828397. The trees are part of a Swedish clonal forestry program grow in a stand situated at Årdala, Sweden, (59°01' N, 16°49' E) [15]. we contacted the land owner, to obtain permission to conduct the study described in this manuscript, and was granted permission by the Forest Manager. The field studies did not involve endangered or protected species. The inoculation and sampling procedures have described in detail in Danielsson et al. [1]. Briefly, three ramets per genotype, and two roots per ramet, were used in the experiment. On one root, a 5 mm diameter woody plug colonized by *H*. *annosum s*.*s*. (isolate Sä 16–4) [16] was attached to an artificial wound on the root surface with Parafilm. The other root on the tree was wounded only and sealed with parafilm. The samples used in this study were harvested at the start of the experiment (0 days post inoculation) and at 5 and 15 days post inoculation (dpi) and preserved in RNAlater (Ambion) for subsequent RNA extraction.

### RNA extraction, cDNA synthesis and sequencing

Total RNA was isolated according to Chang et al. [17]. As previously described [1] poly(A)-RNA from four Norway spruce genotypes (2405, 3178, 3340 and 7398) were purified, amplified and prepared for 454-sequencing on a GS FLX (Roche, 454) at the Norwegian Sequencing Centre (http://www.sequencing.uio.no). Sequence reads and quality scores for sequences were obtained from the Norwegian Sequencing Centre. The reads were deposited at NCBI as SRR346081. Four additional genotypes (5237, 5278, 8397 and 8590) were used for analysis by quantitative reverse transcribed-PCR (qRT-PCR), these samples were also subjected to poly(A)-RNA isolation and mRNA amplification as previously described [1].

### Assembly, annotation and statistical analyses

The bioinformatics and statistics pipeline are outlined in S1 Fig. The retrieved sequences were assembled with the sequence assembler software Newbler v2.6 (Roche) (www.454.com), with default settings for cDNA assembly and the sff-files as input file. The sequence assembly was carried out on the publicly available Bioportal (www.bioportal.uio.no). Sequences from all treatments were assembled into the gene-equivalent isogroups and into the plausible splice variants isotigs. The assembled sequences, excluding the singletons, were used as reference file and were annotated with the software Blast2GO [18]; where the sequences were annotated to blastx homologies and gene ontology (GO) terms. The taxonomic origin of the assembled sequences and singletons were evaluated based on the taxonomic origin of the corresponding blastx homolog by analysing the reference file with MEGAN4 with standard settings [19]

The best reciprocal blast hits of the assembled sequences to Norway spruce gene models and transcripts in the *P*. *abies* 1.0 release of the Norway spruce genome (http://congenie.org/) were identified using blastn 2.2.27 [20]. Blastn was wrapped in a python script which established the best reciprocal matches between the two sequence sets. To identify *H*. *annosum s*.*s*. transcripts the sequences were queried against *H*. *irregulare* sequences in Genbank using blastn [20].

To estimate the relative gene expression between the libraries, individual reads from each library were mapped to the reference file built from the isotigs in the assembly. The mapping was done in Newbler 2.6 at the Bioportal (www.bioportal.uio.no) and count data was generated that was then imported into R v.2.15.0. The count data were normalized toon the overall sum of the counts in each library in the R package DESeq v.1.83 [21]. To reduce the effect of multiple testing on the detection power, filtering independent of the test statistics was applied [22]. Before the test statistic, 40% of the lowest quantile of the overall sum of the counts were removed. The differential expression of genotype, time and treatment dependent expression was tested with the condition based negative binominal distribution test (*nbinomTest*) in DESeq v.1.83 [21] and adjusted for false discovery rate with the Benjamini-Hochberg procedure. The normalized count data underwent variance-stabilizing transformation to homoscedastic data in DESeq and clustered with JMP 9 by Ward’s hierarchical cluster. To determine if there were statistically over-represented GO categories between treatments or clusters we used Fisher's exact test with the Benjamini-Hochberg false discovery rate correction available in the Blast2GO software.

### Verification of gene expression by qRT-PCR

Purified aRNA (1μg) from genotypes 5237, 5278, 8397 and 8590 were reverse transcribed with the iScript cDNA synthesis kit (Bio-Rad). The cDNA synthesis was diluted in deionized water, 1:1, and an aliquot of cDNA equivalent of 25 ng of aRNA was used per 20 μL of PCR reaction using SSoFast EVAGreen Supermix (Bio-Rad) and a final concentration of 0.5 μM of each primer. Primers were designed from isotig sequences using the Primer3 software (http://primer3.wi.mit.edu/) with a melting temperature (Tm) between 58°C and 60°C, and amplicon length between 95–183 bp (S1 Table). The thermal-cycling condition parameters, run on a iQ5 Multicolor Real-Time PCR Detection System (Bio-Rad), were as follows: 95°C for 30 s; 40 cycles of 95°C for 5 s, 58 or 60°C for 20 s. Each run was followed by a melt curve analysis to validate the specificity of the reaction. A linear plasmid standard curve was used to measure the PCR efficiency and primer pairs with efficiency lower than 95% were discarded. Two technical replicates were prepared for each sample.

Transcript abundance was normalized to the reference genes *phosphoglucomutase* [23], *eukaryotic translation initiation factor 4A* (*elF4A*) [24] and *elongation factor 1-α* (*ELF1α*). The relative expression was calculated using the Pfaffl-method [25]. Kruskal-Wallis and Mann-Whitney U-tests were performed on the qRT-PCR data using the GraphPad Prism5 software (GraphPad Inc.).

## Results

### Assembly

Kumar and Blaxter [26] reported that Newbler 2.3 generates assemblies with significantly lower total lengths than newer versions of Newbler. Therefore we chose to reassemble our data with Newbler 2.6 (Roche, www.454.com). We had a limited improvement compared to our previous assembly [1], with an increase in the number of assembled reads and detected genes (isogroups), and a reduction of plausible splice variants compared to with Newbler 2.3 (S2 Table). We did, however, get a reduction in the isotig N50 length.

The taxonomic assignment of the assembled isotigs was estimated with the MEGAN4 software. The absolute majority of the isotigs had plant homologs, and of these most of the blastx hits were to sequences originating from species within the *Pinaceae*, and in particular from *Picea sitchensis* (Table 1). The analysis suggested that very few assembled isotigs were of fungal origin; only 393 isotigs gave a hit to taxa in the fungal kingdom (Table 1).

**Table 1:** Annotation statistics of the assembled transcripts.

Annotation of isotigs	v.2.6
total number of isotigs#	13004
Blast2GO §	
Nr. with BlastX homology	11858
Nr. GO Annotated	7251
MEGAN4 †	
isotigs with plant homologs	9825
-Pinaceae	6367
-*Picea sitchensis* ¥	5829
isotigs with fungal homologs	393
*P*.*abies* 1.0 *	
isotigs with significant hits in the transcript assembly	11908
orphan isotigs¤	1096
-Pinaceae	274
-fungal	205

^#^ Total number of isotigs (putative splice variants of genes) assembled with Newbler 2.6.

^§^ Isotigs annotated with Blast2GO [18]

^†^ Taxonomic assignation with MEGAN4 [19]

^¥^ Isotigs with best hit to *Picea sitchensis* proteins in Genbank

*Reciprocal best blastn hit with the *P*. *abies* 1.0 transcript database [27]

^¤^ Isotigs without a significant hit in the *P*. *abies* 1.0 transcript database, and of these likely *Pinaceae* and fungal genes are reported.

A reciprocal blastn analysis between our dataset and the transcript assembly (Trinity contaminant free) in the Norway spruce genome (*P*. *abies* 1.0 release) [27] showed that 11908 of the assembled isotigs had at least one significant hit in the *P*. *abies* 1.0 transcript assembly (Table 1, S3 Table). Out of the 1096 orphan isotigs, isotigs without significant hits in the Trinity database, some were members of isogroups which had one or more other isotigs variants with significant hits in the transcript assembly and other orphan isotigs had homologs in the *Pinaceae*. Two hundred five orphan isotigs were of fungal origin (Table 1).

### Analysis of the transcriptome

Given the limited depth in our study we filtered away 40% of the lowest quantile of isotigs with mapped read before the test statistic, leaving 7342 isotigs in the analyses of differential expression patterns. Using this dataset we analysed first, the transcriptional changes in Norway spruce in response to inoculation and wounding and secondly a two-way clustering and GO category enrichment analysis was made on the 150 most highly expressed isotigs.

#### Transcriptional changes in Norway spruce in response to inoculation and wounding

**Fig 1:**
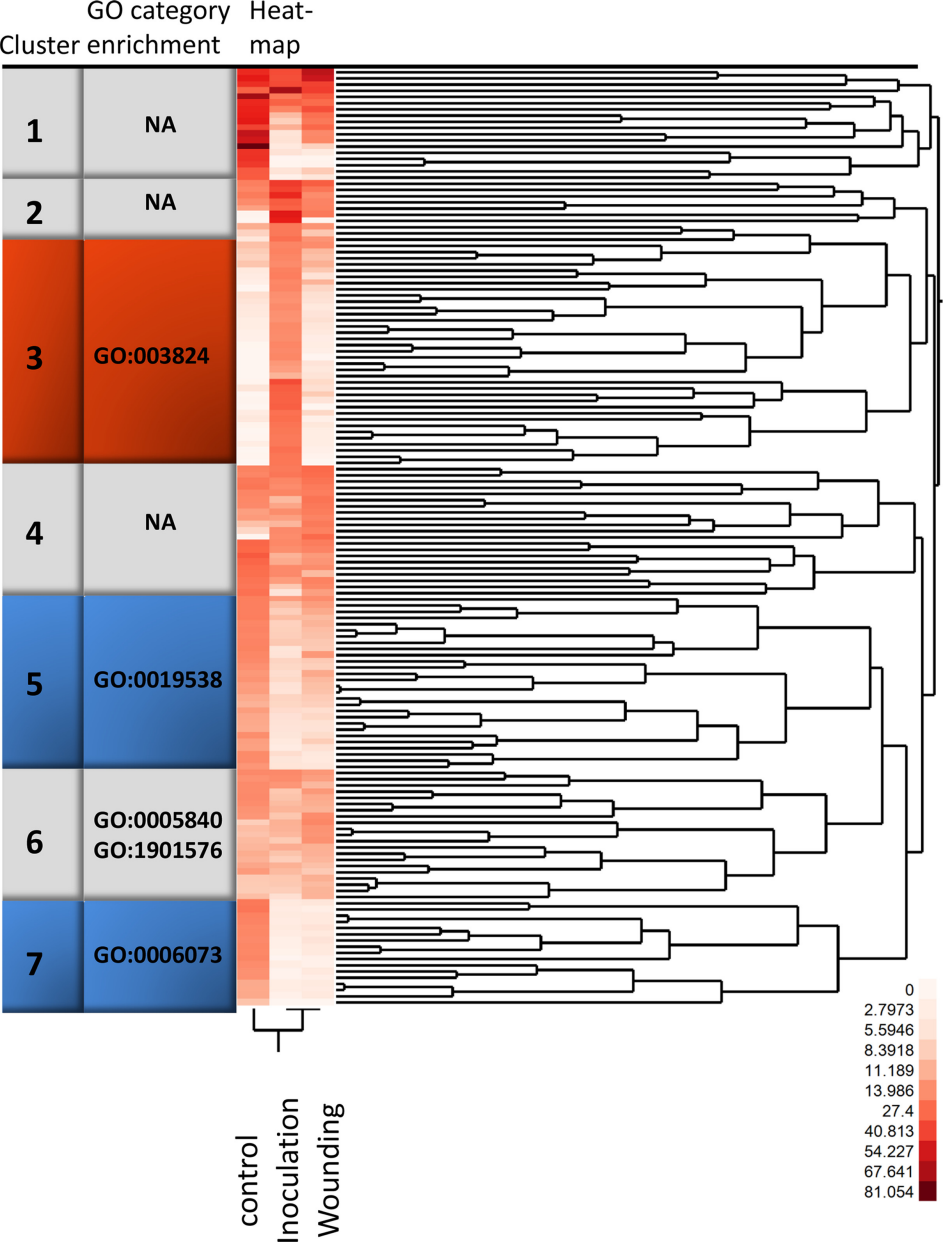
Two-way clustering of the 150 most highly expressed transcripts. A two–way clustering of normalized 454 sequence count data using Ward’s hierarchical cluster algorithm. The increasing intensity of red in the heat map visualise counts from white (no counts in the particular condition) to red. Clusters are indicated by numbers and the enriched GO categories are noted for each cluster. Clusters containing significantly regulated genes are highlighted in colour, with orange for cluster 3, containing genes significantly induced in inoculated samples and blue for clusters with genes more highly expressed in control than in wounding. Please refer to S4 Table for information about specific isotigs found in the clusters.

Compared to untreated bark, transcriptional changes after wounding and inoculation were generally small, and the majority of the assembled isotigs did not show significant changes (*padj* > 0.05) in expression level between the treatments. When comparing the wounded and inoculated samples to the control samples with the *nbinom* test fewer than 40 Norway spruce transcripts were found to be significantly regulated (*padj* < 0.05) in either treatment (Table 2). Ten of the most highly induced genes in the comparison between inoculation and control were found in cluster 3 (Fig 1). A general pattern seen in Table 1 is that genes differentially expressed in response to inoculation were induced while wounding alone resulted in significant down-regulation of genes (Table 2). Only four genes were significantly up-regulated in both comparisons; isotig08156, a protein of unknown function, one *class IV chitinase* genes (isotig01680), one *protein kinase* (isotig00518) and a *thaumatin-like protein* (isotig01982). Several transcripts with similarity to PR-protein gene families were strongly induced in response to inoculation. Besides the already mentioned *class IV chitinase* (isotig01680) and *thaumatin-like protein* (isotig01982) genes, *PR1-like* (isotig05256), *peroxidase* (isotig01055) and *beta–glucosidase* (isotig1778 and isotig01779) genes were differentially expressed.

**Table 2:** Differentially expressed transcripts in inoculation and wounding treatment compared to control.

				Basemean values #		*H*. *annosum* vs control		wounding vs control	
trancript id	Congenie (HC gene)	Hit description	control	*H. annosum*	Wounding	FC (log2) §	padj¥	FC (log2) §	padj¥
isotig08156	MA_111367g0010	protein	0.6	51.2	22.5	6.4	0.001	5.2	0.012
isotig01680	MA_10430424g0010	class iv chitinase	0.4	38.4		6.8	0.003		
contig00509	MA_17102g0020	isoflavone reductase-like protein	0.5	30.7		5.9	0.020		
isotig02279	MA_277805g0010	unknown [Picea sitchensis]	0.0	30.5	8.9	Inf	0.001	Inf	0.008
isotig01779	MA_10394370g0010	beta-glucosidase-like protein	0.0	25.6		Inf	0.002		
isotig04533	MA_276729g0010	desiccation-related protein pcc13-62	0.0	22.6		Inf	0.005		
isotig00518	MA_10427345g0010	protein kinase	0.0	17.2	7.6	Inf	0.003	Inf	0.032
isotig02146	MA_10435706g0010	e-alpha-bisabolene synthase	0.1	16.8		6.9	0.018		
isotig04668	MA_10427432g0020	germin-like protein 2	0.0	16.7		Inf	0.008		
isotig06464	MA_169803g0010	phenylcoumaran benzylic ether reductase-like protein	0.1	15.5		7.2	0.041		
isotig06294	MA_89670g0010	microtubule associated calponin and lim domain containing 2 isoform	0.0	14.1		Inf	0.032		
isotig01778	MA_10394370g0010	beta-glucosidase-like protein	0.0	11.4		Inf	0.018		
isotig06315	MA_75520g0010	atp adp translocator	0.0	11.4		Inf	0.041		
isotig02080	MA_32466g0010	non-symbiotic hemoglobin class 1	0.0	11.1		Inf	0.050		
isotig01982	MA_8772866g0010	thaumatin-like protein	0.0	10.9	5.6	Inf	0.032	Inf	0.037
isotig05256	MA_10428480g0010	pathogenesis-related protein 1	0.0	9.1		7.8	0.048		
isotig05309	MA_10435797g0010	dirigent-like protein	0.0	9.1		Inf	0.045		
isotig06539	MA_106551g0010	protein	0.0	8.1		Inf	0.046		
isotig01014	MA_10437233g0030	protein	0.0	1.3		Inf	0.006		
isotig12672	MA_10431212g0010	conserved hypothetical protein	0.0	0.8		4.3	0.000		
isotig01055	MA_10432865g0020	peroxidase	0.0	0.8		Inf	0.049		
isotig00634	MA_10431025g0010	urea hydro-lyase cyanamide	0.0	0.7		Inf	0.017		
isotig01496	MA_10436415g0010	unknown [Picea sitchensis]	0.0	0.7		4.0	0.006		
isotig10936	MA_158751g0010	bhelix-loop-helix transcription factor	0.1	0.6		2.2	0.049		
isotig02583	MA_689126g0010	protein	0.0	0.6		Inf	0.006		
isotig02712	MA_345316g0010	phosphoribosylformylglycinamidine synthase	0.0	0.6		Inf	0.000		
isotig00099	MA_197296g0010	s-adenosyl methionine synthetase	0.0	0.6		Inf	0.000		
isotig00347	MA_874956g0010	tau class glutathione s-transferase	0.0	0.5		Inf	0.001		
isotig00629	MA_8921185g0010	basic endochitinase-like protein	0.0	0.5		Inf	0.003		
isotig03505	MA_120345g0010	protein	45.9	0.3	0.5	-7.3	0.007	-6.6	0.000
isotig00774	MA_10436472g0010	isoflavone expressed	1.6	0.0	0.0	-Inf	0.032	-Inf	0.008
isotig08082	MA_164918g0010	protein	1.6	0.0	0.1	-Inf	0.014	-4.9	0.012
isotig06080	MA_70143g0010	protein	1.5	0.0	0.0	-Inf	0.014	-Inf	0.003
isotig01200	MA_181562g0010	cytochrome p450	1.5	0.0	0.1	-Inf	0.010	-4.1	0.009
isotig06145	MA_100820g0010	serine-threonine protein plant-	1.4	0.0	0.0	-Inf	0.024	-Inf	0.005
isotig09263	MA_5743523g0010	fad dependent oxidoreductase	1.2	0.0	0.2	-Inf	0.021	-2.3	0.050
isotig08117	MA_40654g0010	gibberellin-regulated protein 3	1.2	0.0	0.1	-Inf	0.044	-3.8	0.037
isotig00281	MA_10107355g0010	unknown [Picea sitchensis]	1.0	0.0	0.2	-Inf	0.044	-2.4	0.037
isotig02227	MA_87055g0010	protein	1.0	0.0		-Inf	0.044		
isotig09117	MA_10428063g0020	rna polymerase ii largest subunit	81.1		8.0			-3.3	0.037
contig00136	MA_120345g0010	dehydrin-like protein	9.5		0.1			-7.5	0.050
isotig05895	MA_2998g0020	unknown [Picea sitchensis]	1.8		0.0			-Inf	0.037
isotig10118	MA_10435769g0010	xylem serine proteinase 1	1.7		0.0			-Inf	0.032
isotig07810	MA_469189g0010	histidine-rich glycoprotein precursor	1.6		0.0			-Inf	0.032
isotig12095	MA_93411g0010	c2 domain-containing protein	1.4		0.0			-Inf	0.050
isotig06890	MA_10155182g0010	harpin-induced protein hin1 from tobacco	1.4		0.0			-Inf	0.037
isotig11557	MA_9937121g0010	protein	1.4		0.0			-Inf	0.032
isotig07764	MA_3726g0010	nbs-containing resistance-like protein	1.2		0.0			-Inf	0.037
isotig00827	MA_125677g0010	cuticular protein 35b	1.2		0.0			-Inf	0.004
isotig09839	MA_16172g0010	protein	1.2		0.0			-Inf	0.032
isotig09226	MA_711804g0010	protein	1.1		0.0			-Inf	0.032
isotig11051	MA_96620g0030	tir p-loop lrr	1.0		0.0			-Inf	0.037
isotig08576	MA_241174g0010	gamma-tocopherol methyltransferase	1.0		0.0			-Inf	0.004
isotig11925	MA_156126g0010	protein	1.0		0.0			-Inf	0.050
isotig01538	MA_836269g0010	thioredoxin h	1.0		0.0			-Inf	0.037
isotig10384	MA_33909g0010	protein	0.9		0.0			-Inf	0.032
isotig09947	MA_568538g0010	extensin-like protein	0.9		0.1			-3.9	0.050
isotig09029	MA_175184g0010	conserved plasmodium protein	0.9		0.1			-2.8	0.050
isotig11630	MA_959749g0010	ecf-family sigma factor	0.0		0.8			Inf	0.005

^#^ BaseMean values indicate the normalized read frequency data.

^§^ The indicated expression values are the average Log 2 fold change values over all genotypes relative the controls (0 dpi) as determined with the condition based negative binominal distribution test (nbinomTest).

^¥^ Padj indicates the P-value after adjustment for false discovery rate with the Benjamini-Hochberg procedure.

**Fig 2:**
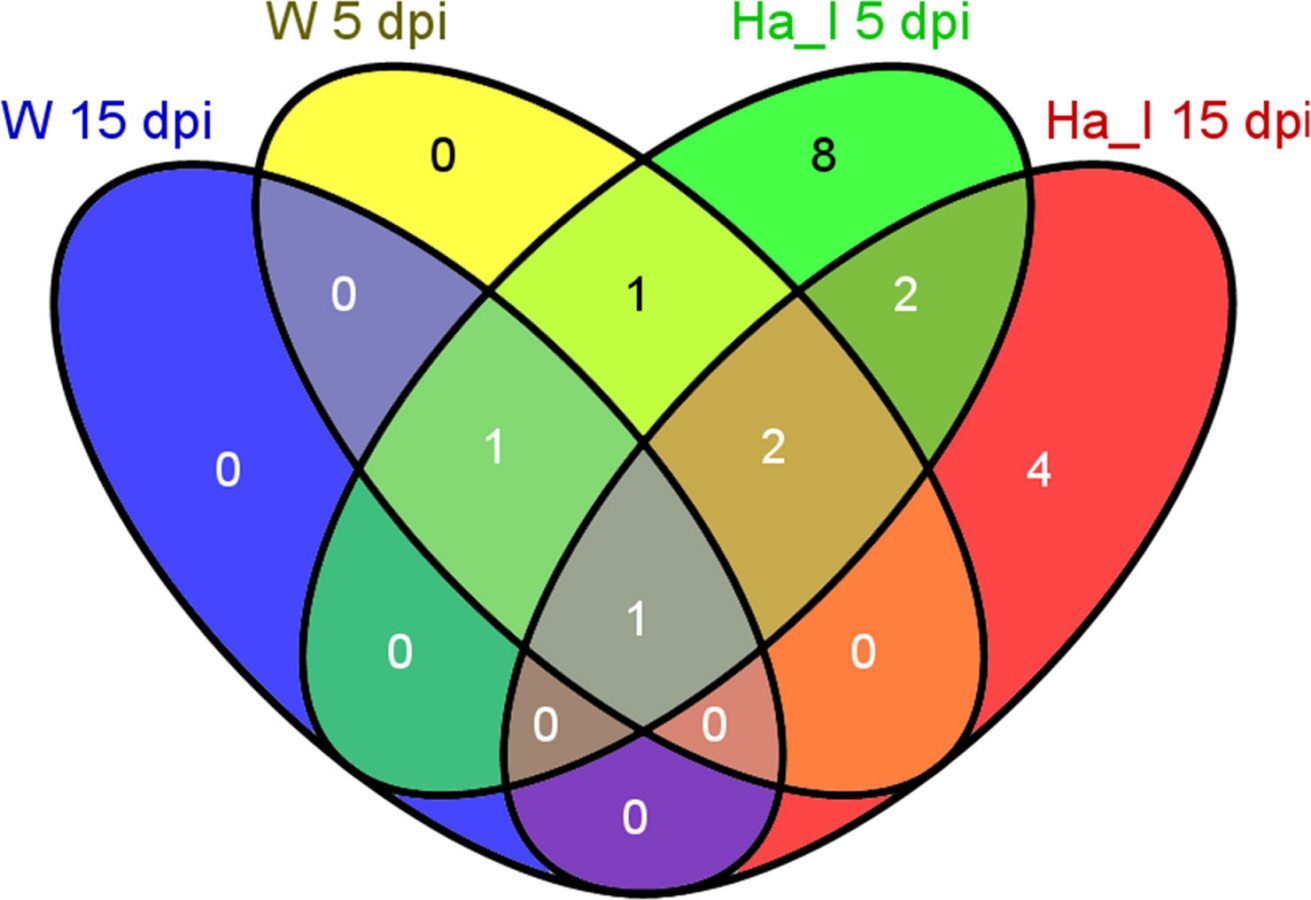
Differentially expressed transcripts in inoculation and wounding treatment relative to the control at different time points. A Venn diagram showing the differentially expressed genes (*P*-value <0.05 after FDR [Benjamini-Hochberg procedure]) at 5 dpi inoculation with *H*. *annosum* (green) or wounding (yellow) and 15 dpi inoculation with *H*. *annosum* (red) or wounding (blue).

Nineteen of the strongly regulated genes (Table 2) were also found to display a significant temporal variation (Fig 2). Irrespective of treatment, more transcripts were differentially regulated at 5 dpi compared to 15 dpi (Table 2). For instance *beta–glucosidase* (isotig1778 and isotig01779) and isotig04668 encoding *PaGLP2*, *Picea abies Germin-like protein 2* [28, 29] were significantly up-regulated in response to inoculation with *H*. *annosum* at 5 dpi. Isotig02279 (MA_277805g0010) encoding a transcript without known function is significantly induced at both time points and treatments (Table 2).

In the comparison between the inoculation and wounding treatments, irrespective of time point, 26 isotigs were found to be significantly up-regulated after inoculation with *H*. *annosum s*.*s*. compared to wounding (Table 3). Fifteen of these differentially expressed isotigs were of Norway spruce origin (Table 3). Five differentially expressed isotigs (isotig06294, isotig06826, isotig08690, isotig01014 and isotig12672) had significant matches in the Norway spruce (v1.0) high confidence gene catalogue, but neither the isotig nor the gene model had significant alignment to any annotated protein in Genbank. A transcript differentially expressed between inoculation and wounding but not detected in the previous analyses, was isotig00380. This transcript has similarity to *12-oxophytodienoic acid–reductase* and it was induced 8 times in inoculated samples compared to wounded samples (Table 3). No significant differences in expression patterns between wounding and inoculation could be detected when the two time points were analysed separately (data not shown).

**Table 3:** Differentially expressed transcripts between inoculation and wounding treatments.

				Basemean values #			
trancript id	Congenie (HC gene)	*H. irregulare*	Hit description	*H. annosum*	Wounding	FC (log2)§	padj¥
isotig00380	MA_46456g0010		12-oxophytodienoic acid-reductase	14.7	0.1	8.1	0.010
isotig06294	MA_89670g0010		uncharacterized protein	14.1	0.1	8.0	0.003
isotig01778	MA_10394370g0010		beta-glucosidase-like protein	11.4	0.1	7.7	0.003
isotig08690	MA_17594g0010		protein	10.8	0.1	6.2	0.014
isotig08958	MA_825689g0010		mfs family major facilitator transporter	9.9	0.2	6.0	0.045
isotig04668	MA_10427432g0020		germin-like protein 2	16.7	0.3	6.0	0.003
isotig01779	MA_10394370g0010		beta-glucosidase-like protein	25.6	1.8	3.8	0.018
isotig02712	MA_345316g0010		phosphoribosylformylglycinamidine synthase	0.6	0.1	3.6	0.006
isotig00099	MA_197296g0010		s-adenosyl methionine synthetase	0.6	0.1	3.5	0.010
isotig00347	MA_874956g0010		tau class glutathione s-transferase	0.5	0.0	Inf	0.007
isotig01014	MA_10437233g0030		protein	1.3	0.0	Inf	0.014
isotig01496	MA_10436415g0010		unknown [Picea sitchensis]	0.7	0.0	Inf	0.007
isotig02790	MA_12996g0010		peptidase m28 family protein	1.1	0.0	Inf	0.026
isotig06826	MA_89670g0010		hypothetical protein	7.6	0.0	Inf	0.019
isotig12672	MA_10431212g0010		conserved hypothetical protein	0.8	0.0	Inf	0.000
isotig03463		ETW80796.1	hypothetical protein HETIRDRAFT_46394	0.7	0.2	2.2	0.003
contig00252		ETW77667.1	NAD(P)+-dependent aldehyde dehydrogenase	7.5	0.0	Inf	0.043
isotig03399		ETW81018.1	protein	13.7	0.0	Inf	0.007
isotig04483		ETW77095.1	delta-12 fatty acid desaturase	49.6	0.0	Inf	0.000
isotig04522		ETW86881.1	mitochondrial carrier protein	12.2	0.0	Inf	0.003
isotig04652		ETW77095.1	delta-12 fatty acid desaturase	10.6	0.0	Inf	0.006
isotig05435		ETW83498.1	2OG-Fe(II)oxygenase superfamily	10.2	0.0	Inf	0.035
isotig05463		ETW77100.1	delta-12 fatty acid desaturase	9.9	0.0	Inf	0.005
isotig09146		ETW83069.1	weakly similar to hypothetical protein	14.7	0.0	Inf	0.003
isotig10242		ETW81403.1	weakly similar to CRISPR-associated protein 1	10.5	0.0	Inf	0.010

^#^ BaseMean values indicate the normalized read frequency data.

^§^ The indicated expression values are the average Log 2 fold change values over all genotypes relative the controls (0 dpi) as determined with the condition based negative binominal distribution test (*nbinomTest*).

^¥^ *Padj* indicates the *P*-value after adjustment for false discovery rate with the Benjamini-Hochberg procedure.

#### GO category enrichment among highly expressed genes

Among the 150 most expressed transcripts (basemean > 7.7) after filtering of the transcripts 75% had blastx homologs in *Picea spp*. and 3% had blastn homologs in the *H*. *irregulare* genome. Norway spruce transcripts associated with translation (GO:0006412), ribosome (GO:0005840), cellular biosynthetic process (GO:0044249) and gene expression (GO:0010467) were enriched (Fisher's Exact Test; *P* <0.05 after correction for multiple testing) compared to the data set as a whole. A two-way clustering of these most highly expressed transcripts displayed seven clusters (Fig 1, S5 Table). Cluster 6, together with clusters 1, 2, and 4, showed similar expression levels between treatments. Fisher's Exact Test for enrichment of any GO category (*P* <0.05 after correction for multiple testing) showed an overrepresentation of transcripts in GO categories ribosome (GO:0005840) and organic substance biosynthetic process (GO:1901576) in cluster 6. Clusters 1, 2 and 4 showed no enrichment of GO categories.

Cluster 3, with the highest levels of transcript accumulation in inoculated bark (Fig 1), had an overrepresentation of transcripts associated with GO:0003824 (catalytic activity). The transcript accumulation patterns in clusters 5 and 7 indicated higher expression levels in untreated bark (Fig 1). Fisher's Exact Test indicated that Cluster 5 contained transcripts associated with protein metabolic process (GO:0019538) and cellular glucan metabolic processes (GO:0006073) was overrepresented in cluster 7.

#### qRT-PCR validation of expression patterns of Norway spruce transcripts

**Fig 3:**
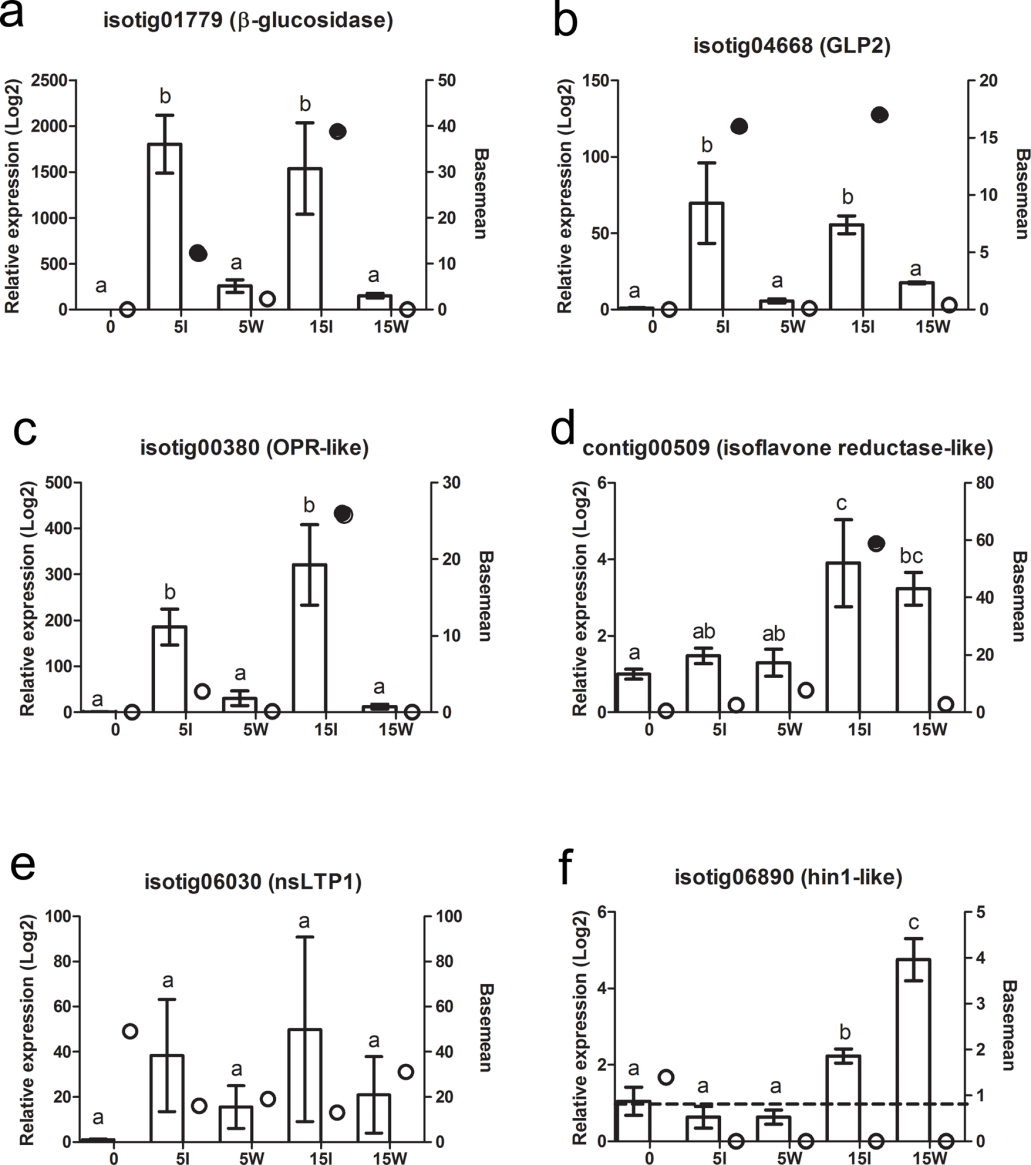
Expression pattern of selected transcripts in an independent Norway spruce material compared to the RNAseq frequency data. Isotig01779, *beta-glucosidase* (a); isotig04668 *GLP2* (b); isotig00380, *OPR-like* (c); contig00509, *isoflavone reductase-like* (d); isotig06030, *ns-LTP* (e) and isotig06890, *hin1-like* (f). The columns indicate relative expression levels over the control, determined with qPCR, for each time point and treatments. The bars indicate the standard error (SE) and superscript letters indicate significant differences between treatments (*P*<0.05, Kruskal-Wallis test with Dunn´s post test), N = 3. The circles indicate the basemean data reported by DEseq, where shaded symbols were significantly different from un-treated bark.

A small subset of transcripts was selected for verification of the RNAseq data by qPCR analysis in independent Norway spruce genotypes. As predicted by the RNAseq data, isotig01779 and isotig04668 showed higher expression in the inoculated samples than wounded alone at both 5 and 15 dpi (Fig 3a and 3b). RNAseq data predicted a significant induction of contig00509 and isotig00380 in response to inoculation at 15 dpi; this was was observed for both transcripts (Fig 3c and 3d). However, isotig00380 also showed significant induction at 5 dpi (Fig 3c) and contig00509 was induced in response to wounding at 15 dpi (Fig 3d). According to the RNAseq data, isotig06030 (*type1 nsLTP*) was highly expressed but showed no differential expression between treatments, which was validated by qPCR (Fig 3e). Isotig06890 (*Hin1-like*) was expected to be expressed at low levels but without significant regulation, and was corroborated by qPCR data at 5 dpi. However at 15 dpi significant induction were seen in both wounded and inoculated samples (Fig 3f).

### *Heterobasidion annosum s*.*s*. transcripts accumulating in inoculated bark

Among the significantly induced transcripts, ten transcripts showed significant alignment to *H*. *irregulare* TC32-1 sequences in Genbank (Table 3). Three *delta-12 fatty acid desaturase* transcripts i.e. isotig04483, isotig04652, isotig05463 (ETW77095.1, ETW77100.1 ETW81752.1 respectively) were found among the significantly induced transcripts. Other significantly induced transcripts were isotig10242 *(weakly similar to CRISPR-associated protein 1*, *ETW81403*.*1)*, isotig04522 (*mitochondrial carrier protein*, ETW86881.1 [30]) and isotig05435 (*Clavaminate synthase-like*, ETW83498.1). Among the predicted *Heterobasidion* transcripts isotig03463 is the only transcript that had any mapping reads from the wounding treatment. The corresponding *H*. *irregulare* gene (ETW80796.1) encodes a highly conserved protein present in all Dikarya. The two isotigs/genemodels isotig03399/ETW81018.1 and isotig09146/ETW83069.1 showed no significant alignments outside the taxon of *Heterobasidion* in Genbank but were highly expressed in inoculated samples (basemean 13.7 and 14.7, respectively).

Thereafter, we searched for expressed transcripts (Basemean >1) with a significant alignment to the *H*. *irregulare* gene models available in Genbank and 54 sequences specifically expressed in inoculation treatments were identified (S6. Table). Most of the identified transcripts match genes in primary- (GO:0044238), single-organism- (GO:0044710), cellular- (GO:0044237) and organic substance- (GO:0071704) metabolic processes. The most highly expressed transcript was a *delta-12 fatty acid desaturase* (isotig04483) (Table 3, S3 Table). Probes corresponding to 49 of the identified *H*. *irregulare* gene models were included on the micro-array made by Olson and co-workers [14] (S5 Table). Only seven transcripts identified in the *H*. *annosum s*.*s*.-Norway spruce bark interaction were among the 250 most highly expressed *H*. *irregulare* genes in necrotic bark tissue after the filtering for potential cross-hybridisation by Pine transcripts [14]. However, generally the *H*. *irregulare* homologs to the identified 49 *H*. *annosum s*.*s*. transcripts showed very high relative expression after inoculation in Pine (relative expression values > 10000) on the microarray (S5 Table). Irrespective of potential cross-hybridization with Pine, seven gene models showed higher relative expression on necrotic bark than in pure culture of *H*. *irregulare* (S5 Table) and high levels of expression in the *H*. *annosum s*.*s*.-Norway spruce interaction. Two of these were isotig10242 *(weakly similar to CRISPR-associated protein 1*, *ETW81403*.*1)* and isotig05435/ETW83498.1 (*Clavaminate synthase-like*) (Table 3, S4 Table).

## Discussion

The main objective of this study was to characterize the transcriptional changes associated with induced defence responses in Norway spruce to the necrotrophic root rot fungus *H*. *annosum s*.*l*. however, the statistical analyses of the transcriptome data revealed relatively few differentially expressed transcripts. One obvious reason to this observation is that with smaller datasets, such as ours, the accuracy in the analyses of genes with a low expression level decreases more than the accuracy in the analyses in genes with higher expression levels [31]. In order to improve the accuracy we filtered our dataset to remove the 40% least abundant transcripts, even so only a handful of genes were differentially expressed between inoculation and wounding treatments. However, also previous work report an extensive overlap in the responses to wounding and *H*. *annosum s*.*l*. inoculation in this material [1] and in other Norway spruce materials [4, 5, 7, 8].

Several of the genes that were differentially expressed between inoculated bark and control have previously been reported to be strongly up-regulated in response to biotic stress in *Pinaceae* e.g. *class IV* and *II chitinases* [32], *PR1-like*, [4] *peroxidase* [33] *germin-like protein* [28], *thaumatin-like protein* [34] and *dirigent* protein [35]. Most of these were also found in cluster 3, with the most highly expressed transcripts in inoculated bark.

A noteworthy observation is the apparent repression of 29 transcripts in wounded bark compared to the control; most of these transcript show very low relative expression values already in the control and must be treated with caution. However three of the transcripts are highly expressed in the control, of which encodes putative *dehydrin* genes (isotig03505 and contig00136). Dehydrins play a fundamental role in plant response and adaptation to abiotic stresses [36–38]. They accumulate typically in maturing seeds or are induced in vegetative tissues following salinity, dehydration, cold and freezing stress. Despite all the research on dehydrins, their regulation in relation to their function is not fully understood. It has been described that different members of the *dehydrin* gene family in Norway spruce and Maritime pine (*Pinus pinaster*) differential regulation in development and in response to abiotic stress [37, 39, 40]. At least one *Picea dehydrin* gene has been reported to respond to wounding, MeJA and ethylene treatments [36]. This indicates large functional differences between different *dehydrin* gene family members [40] and an overlap in the transcriptional responses to drought and wounding in the *dehydrin* gene family. Thus, it is possible that signalling cues other than drought stress can induce the suppression of the *dehydrin* genes isotig03505 and contig00136 after wounding.

Fifteen Norway spruce transcripts where differentially expressed between inoculation and wounding. All of the transcripts were more highly expressed after inoculation compared to wounding, which also agrees with our previous observations in other materials [4], namely that responses are more aggravated after inoculation than wounding. Three genes, not previously associated with defence to *H*. *annosum s*.*l*., showed specific induction in response to inoculation; one *oxophytodienoic acid–reductase (OPR)*, one *beta–glucosidase* and one *GLP2 (isotigs* 00380, 01778, 01779 and 04668, Fig 3a–3c). OPRs catalyze the reduction of double bonds adjacent to an oxo- group in a,b-unsaturated aldehydes or ketones. Many OPRs are part of the octadecanoid pathway that converts linolenic acid to jasmonic acid (JA), but 12-oxo-phytodienoic acid can also act as a mediator in the defence against necrotrophic pathogens [41–43]. The induction of isotig00380 *(OPR)* in inoculated samples compared to wounded samples (Fig 3c), fits with the observation that JA-mediated signalling is a central pathway in the defence against *H*. *annosum s*.*l*. and highly induced in response to the fungus [4, 8].

Two *germin-like* genes *GLP1* and *GLP2* have previously been reported to be highly induced in Norway spruce seedlings in response to inoculation with *Ceratobasidium bicorne* [28, 29]. Our observation that *GLP2* is specifically induced in response to *H*. *annosum* inoculation (Fig 3b) could indicate that at least *GLP2* may respond to particular signalling cues from biotic stressors not present in the wounding treatment. However it has been reported that drought may influence the pathogen-induced expression *GLP2* expression patterns and that the induction patterns in response to *C*. *bicorne* varies between roots and shoots of young in Norway spruce seedlings [29]. Thus further studies of the expression pattern of *GLP2* in response to *H*. *annosum* are needed.

Two putative splice variants of the *beta–glucosidase* gene MA_10394370g0010, isotig1778 and isotig01779, were both highly induced in inoculated bark (Tables 2 and 3). Beta–glucosidases, with capacity to hydrolyze coniferin (coniferyl alcohol 4-*O-*glucoside) into coniferyl alcohol, which can be used for cell wall reinforcement, are known from cambium and primary xylem in *Pinus* and *Picea* [44, 45]. It is interesting that the induction of these transcripts were specific to inoculated materials (Fig 3a), as the phenylpropanoid pathway and lignification are induced in response to both inoculation and wounding [4, 5]. The induction of this *beta-glucosidase* gene could indicate that a local release of precursors for lignification are activated to control the spread of *H*.*annosum s*.*l*. in the cambium and phloem.

A second objective was to identify *H*. *annosum s*.*s*. genes expressed in the pathogen´s interaction with bark of Norway spruce. However, the proportion of fungal reads was very low. Fewer than 400 putative *Heterobasidion* transcripts were assembled and of these a fourth was retained after filtering of the expression data. The vast majority of these expressed *H*. *annosum s*.*s*. transcripts encodes genes in the metabolic machinery sustaining fungal growth. This observation is supported by the comparison with the expression profiles of *H*. *irregulare* in necrotic pine [14] which showed that the corresponding *H*. *irregulare* genes generally were highly expressed both in necrotic bark and pure culture. The identification of three *Heterobasidion delta-12 fatty acid desaturases* (*delta-12 FADs*), which were highly expressed in inoculated bark are examples of such central genes probably sustaining fungal growth. Delta-12 FADs are known from many fungal species, and produce the major components of fungal cell membranes [46–50]. It has been shown that *Aspergillus nidulans* and *A*. *parasiticus delta-12*-*FAD* mutants have reduced growth, conidiation and impaired host colonization [51, 52]. The protein encoded isotig05435/ETW83498.1 (*Clavaminate synthase-like*), could be involved in secondary metabolite production as Clavaminate synthases belong to the non-heme iron, alpha-ketoglutarate-dependent oxygenases and catalyse the production of antibiotic compounds [53, 54].

In the comparison between our 454-dataset and the *H*. *irregulare* microarray analysis described by Olson and colleagues [14] it is obvious that most of the *H*. *irregulare* genes homologous with the *H*. *annosum s*.*s*. genes in our experiment were filtered out, due to cross-hybridisation with Pine in the microarray study. A handful of genes present in both datasets showed higher relative expression levels in bark compared to pure culture on the *H*. *irregulare* microarray [14] possibly representing *H*. *annosum s*.*l*. virulence factors. Three candidates are isotig10204/ETW808090.1 (*Formate dehydrogenase*), isotig12111/ETW81845.1 (hypothetical protein) and isotig10242/ *ETW81403*.*1* (containing the pfam domain PF11327). The two first showed differential expression on the microarray and expression in the 454-data set, while isotig10242 *(weakly similar to CRISPR-associated protein 1*, *ETW81403*.*1)*, showed high expression levels in *H*. *annosum s*.*s*. Isotig10204 has the GO annotation GO:0043581 (mycelium development), suggesting that it, just like the *delta-12 FAD* genes, is involved in host colonization. The function of isotig12111/ETW81845.1 and isotig10242/ *ETW81403*.*1* cannot be accurately predicted using sequence similarity and GO annotation as neither transcript has GO annotations. Further studies of these two candidates are needed to establish their role in virulence.

Even with a small data set like ours, novel Norway spruce transcripts without significant alignment to any previously annotated protein in Genbank but present in the *P*.*abies* 1.0 High confidence gene catalog were identified. Predicting a role for these gene models in Norway spruce defence responses. Thus a more complete survey of the transcriptional responses in the interactions between Norway spruce and its major pathogen *H*. *annosum s*.*l*. would probably provide a better understanding of gymnosperm defence.

## Supporting Information

S1 Fig The bioinformatics and statistics pipeline. (PDF)

S1 Table qRT-PCR primers and sequences. (XLSX)

S2 Table Transcriptome assembly and annotation statistics. (XLSX)

S3 Table Reciprocal BlastN. (XLSX)

S4 Table The 150 most highly expresses isotigs included in the cluster analysis and their cluster. (XLSX)

S5 Table *H*. *annosum* transcripts and a comparison with *H*.*irregulare* gene expression. (XLSX)
