# Supplementary figures and images for: Transcriptional Responses Associated with Virulence and Defence in the Interaction between Heterobasidion annosum s.s. and Norway Spruce
Source: PLoS One. 2015 Jul 7;10(7):e0131182. doi: 10.1371/journal.pone.0131182 (PMC4495060; doi:10.1371/journal.pone.0131182)

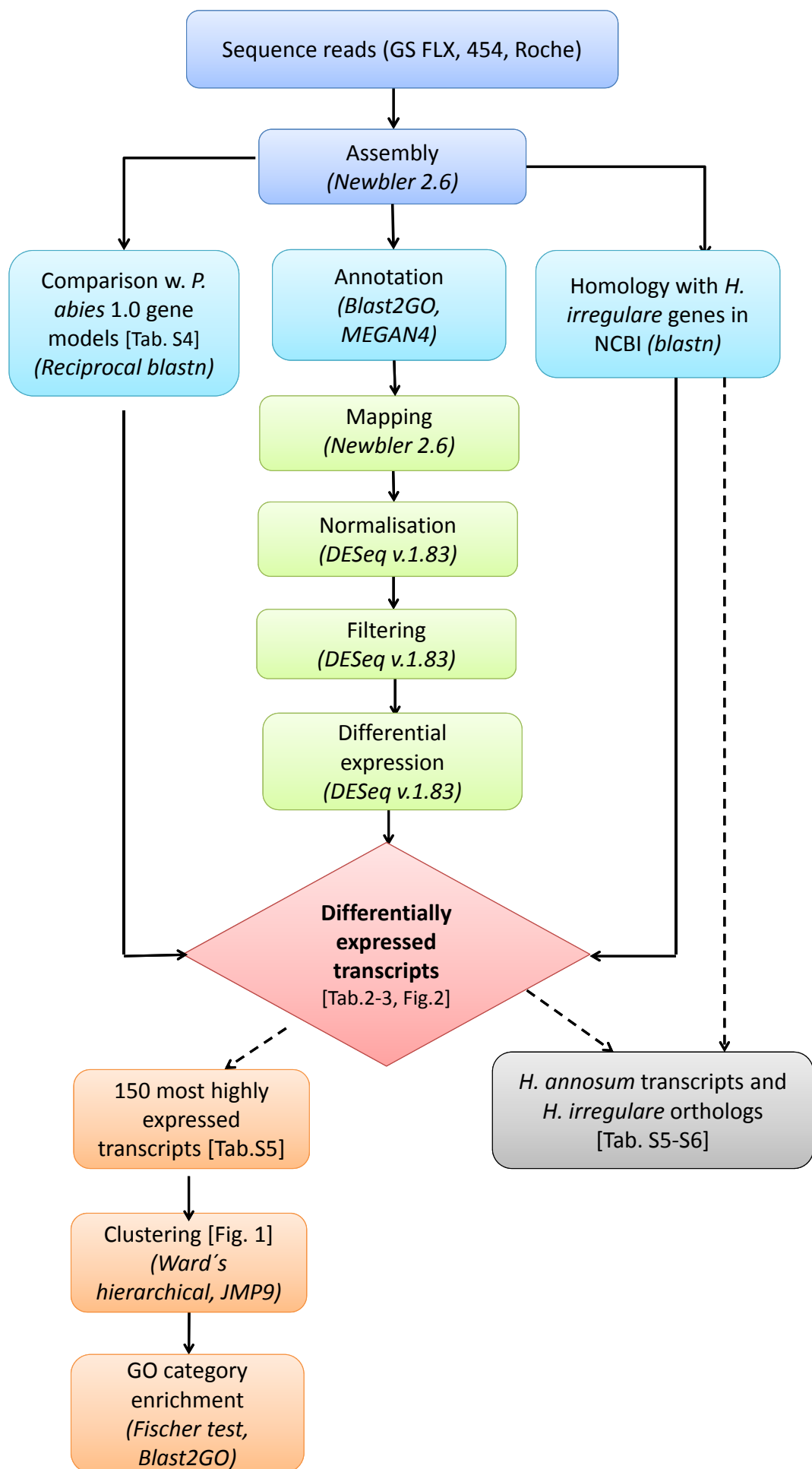

Supplement: S1 Fig — (PDF) [file pone.0131182.s001.pdf]
